# Supplementary material for: Rapid turnover of life-cycle-related genes in the brown algae
Source: Genome Biol. 2019 Feb 14;20:35. doi: 10.1186/s13059-019-1630-6 (PMC6374913; doi:10.1186/s13059-019-1630-6)
Supplement: Supplementary file 3 — Protein alignments, corresponding Gblocks html file and CDS sequences for the lineage conserved gametophyte-biased genes. (ZIP 2109 kb) [file 13059_2019_1630_MOESM3_ESM.zip › Con_GA_alignments/Ec-25_003780.aa_ali.fasta-gb.htm]

Ec-25\_003780.aa.fasta.trans.aa\_ali.fasta


## Gblocks 0.91b Results

Processed file: **Ec-25\_003780.aa.fasta.trans.aa\_ali.fasta**  
Number of sequences: **2**  
Alignment assumed to be: **Protein**  
New number of positions: **320** (selected positions are underlined in blue)

```
                         10        20        30        40        50        60
                 =========+=========+=========+=========+=========+=========+
Ec-25_003780     MVKANKIGGRGRRKRRKQDEDAAATRGKGVYAQPKLSPDQYVGDFDEIPIGLPGSAPCSL
MSTRG.40373.1    ---------------------------------------------DEIPMGLPGSVPCGP
                                                              ###############


                         70        80        90       100       110       120
                 =========+=========+=========+=========+=========+=========+
Ec-25_003780     RICCCCLNAGEFETISNREEESEFPCCSCLPREKKKKLSNNPKGNEGDGTASQGSTPEQG
MSTRG.40373.1    RTCCCCLNAGEFETISKREEPSRAPCC---PRPRRNKDSSQ-RGDAG--TAAGNSNPEKI
                 ###########################                      ###########


                        130       140       150       160       170       180
                 =========+=========+=========+=========+=========+=========+
Ec-25_003780     QQSAG--DTDDDELEPDRWGRVPLGRKGWRICLTMGFLPFALPCRASEQHKQLTELYPVE
MSTRG.40373.1    RQAEATATASDHLAEHNRRGRVPLGEEGWRVCLTMLCFPWALPWRACTQHKNITELYPVE
                 ##        ##################################################


                        190       200       210       220       230       240
                 =========+=========+=========+=========+=========+=========+
Ec-25_003780     STLKNKSGVCGRIKAAVFFPWMLAKHVAFLRERRWADELRYPWQTRAAWTVAGRPPSALF
MSTRG.40373.1    STTTQKSGLCGRIKATVLFPWMLAKHVAFVRERRWADELRYPWQTRAVWTVAGRPPSALF
                 ############################################################


                        250       260       270       280       290       300
                 =========+=========+=========+=========+=========+=========+
Ec-25_003780     KILVLGDNGVGKTALARALCGKG-FLPEYSPDTAVSVSARSFSRDKFPPAVLEIWDIPAS
MSTRG.40373.1    KVLILGDNGVGKTALARALCGKGGLLPEYTPGTAVSVSARSFSRENYPPAVLEIWDVPAS
                 #######################  ###################################


                        310       320       330       340       350       360
                 =========+=========+=========+=========+=========+=========+
Ec-25_003780     AKTSLGPEIAAETSCALLLLDLDDRNSFRVAKRWQSLVTEACSGSKARPPHFIVVGTKID
MSTRG.40373.1    AKTTVGTEMVTETSCALLLLDLDDRNSLRVAKRWQALITEACSGSKEGPPQFIVVGTKID
                 ############################################################


                        370       380       390       400       410       420
                 =========+=========+=========+=========+=========+=========+
Ec-25_003780     VSPASRCCFLKGVRGWCEEQGYPLLFCTSFAGEGIMGILREVVVTCFERHPPPAVLKDLY
MSTRG.40373.1    VTPASNCAFFKEAQLWCESNGAPLLFCTSFTGEGIMGX----------------------
                 #####################################                       


                 
                 ===
Ec-25_003780     LAV
MSTRG.40373.1    ---
```

```
Parameters used
Minimum Number Of Sequences For A Conserved Position: 2
Minimum Number Of Sequences For A Flanking Position: 2
Maximum Number Of Contiguous Nonconserved Positions: 8
Minimum Length Of A Block: 10
Allowed Gap Positions: None
Use Similarity Matrices: Yes
```

```
Flank positions of the 4 selected block(s)
Flanks: [46  87]  [110  122]  [131  263]  [266  397]  

New number of positions in Ec-25_003780.aa.fasta.trans.aa_ali.fasta-gb:  320  (75% of the original 423 positions)
```
